# The Role of Livestock Antibiotic Use in Microbiota Dysbiosis and Neuroinflammation

**DOI:** 10.3390/antibiotics14060608

**Published:** 2025-06-15

**Authors:** Serena Silvestro, Carmelo Biondo, Angelina Midiri, Borrello Lucia, Giuseppe Mancuso

**Affiliations:** 1 IRCCS Centro Neurolesi “Bonino-Pulejo”, Via Provinciale Palermo, Contrada Casazza, 98124 Messina, Italy; serena.silvestro@irccsme.it; 2 Mycology Laboratory, Department of Human Pathology, University of Messina, 98125 Messina, Italy; cbiondo@unime.it (C.B.); amidiri@unime.it (A.M.); 066867@polime.it (B.L.)

**Keywords:** livestock, antibiotic, antimicrobial resistance, One Health, multidrug-resistant, microbiota dysbiosis, neuroinflammation

## Abstract

Antibiotic overuse in livestock is a major concern, as it contributes to the emergence of antibiotic resistance and may adversely affect both animal and human health. One important consequence is its impact on the gut microbiota, a complex microbial ecosystem essential for maintaining host health. A growing body of research highlights the critical role of a balanced gut microbiota in maintaining the integrity of the gut-microbiota–brain axis, a bidirectional communication network between the gastrointestinal tract and the central nervous system (CNS). Antibiotics introduced through the food chain and the environment can disrupt microbial balance, leading to dysbiosis and systemic inflammation. In this context, the concept of “One Health” is emphasized, which recognizes the deep interconnection between the health of humans, animals, and the environment to address the global problem of antibiotic resistance. Several animal studies highlight how dysbiosis can induce neuroinflammation and potentially damage the gut–brain barrier. This review explores the mechanisms by which antibiotic use in livestock alters the gut microbiota and compromises the gut-microbiota–brain axis integrity, outlining the implications for public health and the possible link with neurodegenerative conditions.

## 1. Introduction

Antibiotics are widely used in livestock not only to treat sick animals, but also to prevent disease and promote growth. However, this widespread and often excessive use has contributed to the emergence of antibiotic-resistant bacterial strains, organisms that are increasingly difficult, and in some cases impossible, to treat. Antibiotic resistance is becoming a serious global health crisis, undermining the effectiveness of life-saving treatments and putting millions of lives at risk. Each year, more people and animals suffer from infections that no longer respond to standard antibiotics. Experts warn that this trend will only accelerate without urgent and coordinated action [1]. Projections indicate that deaths caused by infections could rise from 700,000 per year in 2014 to as many as 10 million by 2050 [2]. The economic impact could be equally devastating, with an estimated $100 trillion in healthcare costs and lost productivity. Even in countries that have implemented strict policies to limit antibiotic use, resistance continues to rise, showing that the problem extends beyond national borders and requires a coordinated global response [3].

The spread of antimicrobial resistance (AMR), both locally and globally, is driven by several factors, including the misuse of antibiotics in humans and livestock, environmental contamination, and inadequate infection control policies [4,5]. Irresponsible use of antibiotics in humans promotes the selection and spread of resistant bacteria and antibiotic resistance genes (ARGs), which can be transmitted between individuals via direct contact, consumption of contaminated food and water, or environmental surfaces. Similarly, antibiotic use on livestock farms contributes to the emergence of bacterial resistance, which can reach humans through contact with animals, consumption of animal products, or environmental dissemination via livestock farming [1,6].

In animal farming, antibiotics are frequently administered for non-therapeutic purposes such as growth promotion and disease prevention. These antibiotics are often detected in the gastrointestinal tracts of animals at sub-lethal concentrations, which selectively favor the proliferation of resistant bacterial populations. As a result, ARGs accumulate and persist in livestock waste, spreading to the surrounding environment, including soil and water systems [7,8].

There is growing concern about the potential transfer of ARGs from livestock to humans, which may occur through direct contact, environmental exposure, or the food chain. While a direct correlation between ARGs from animal farming and their acquisition by the human gut microbiome has not yet been fully established, evidence suggests that agricultural workers and nearby communities may present a high risk of exposure to resistant bacterial strains. The ARG persistence in the environment may also contribute to the growing number of resistant clinical infections, which are becoming increasingly difficult to treat.

New research is shedding light on a troubling aspect of antibiotic use: it is not only the risk of bacterial resistance, but also their impact on our internal balance. Antibiotics can profoundly alter the gut microbiota, creating an imbalance that is not limited to the digestive tract, but extends throughout the body [9,10,11]. This can fuel chronic inflammation and, according to some studies, may even contribute to the development of neurodegenerative diseases such as Alzheimer’s [12], Amyotrophic Lateral Sclerosis [13], and Parkinson’s [14]. These emerging links between antibiotics, inflammation, and neurological problems suggest important questions about the long-term effects of antibiotics on human health, highlighting the need for targeted therapeutic strategies and more informed use of antimicrobials.

This review examines the mechanisms by which antibiotic use in livestock farming can alter the gut-microbiota–brain axis, with potential effects on human health. Transmission of resistance genes mediated by mobile genetic elements is also analyzed. Particular attention is paid to the complex interaction between the gut microbiota and the brain. This interaction, in addition to playing a protective role, may also contribute to the onset and maintenance of a chronic intestinal inflammatory state, the spread of systemic inflammation, and the impairment of physiological barriers. A central concept in this context is dysbiosis, defined as an imbalance or disruption in the composition of the gut microbiota, has been associated with various pathological conditions including inflammatory, metabolic, and neuropsychiatric disorders. These mechanisms represent potential risk factors for the development of neurodegenerative processes and behavioral disorders. Finally, the need for a coordinated and multidisciplinary approach to address this problem on a global scale is emphasized.

## 2. Use of Antibiotics in Livestock and Resistance Development

The excessive and improper use of antimicrobial drugs in livestock farming represents a major challenge in veterinary medicine, significantly contributing to the development of AMR. This issue is not confined to animal health but extends to human healthcare, where multidrug-resistant foodborne pathogens are becoming increasingly common [15].

Key factors influencing AMR development include drug formulation, dosage, treatment frequency, and therapy duration. Evidence from theoretical models, laboratory experiments, and clinical studies suggests that shortening the duration of subtherapeutic drug concentrations in infected tissues can help mitigate resistance development.

Historically, efforts have focused on combating specific pathogenic organisms. However, it is crucial to acknowledge that antimicrobial use also affects the host’s normal microbiota. Over time, this impact becomes just as significant as direct pathogen resistance selection, as horizontal gene transfer enables nearly all pathogenic bacteria to acquire and spread resistance traits.

Other key factors driving AMR include microbial population density, host age, immune system function, and the degree of human–animal interaction. In livestock, resistance patterns have evolved across different species, reflecting shifts in antibiotic use practices [16].

Livestock farming intersects with three major global concerns: public health, environmental sustainability, and socioeconomic equity. There is a growing consensus that antibiotic use in animal husbandry significantly contributes to the emergence of antibiotic-resistant infections in humans [17].

Antibiotic resistance is one of the most pressing global health challenges and is a clear example of how interconnected human, animal, and environmental well-being are [18]. Bacteria such as *Escherichia coli*, *Salmonella*, and *Campylobacter* can develop ARGs and spread among animals, people, and the environment through various channels, including antibiotic residues and mobile genetic elements (MGEs) [19]. Excessive antibiotic use in agriculture, livestock, and human medicine has further intensified this crisis, making it increasingly urgent to adopt a coordinated and comprehensive approach to counter its effects [20]. As a result, these drugs constantly accumulate in the gastrointestinal tract of livestock at low and sub-lethal concentrations, slowing the growth of susceptible bacteria and creating a favorable environment for the development of ARGs. This selective pressure increases the prevalence of resistant bacterial populations in the digestive system of animals [16]. When ARGs spread to the surrounding environment, antibiotic resistance becomes an environmental pollution problem, turning these genes into contaminants of increasing concern [21]. For example, resistant bacteria in the intestinal tract of animals are excreted through feces, releasing ARGs into soil and water resources [7]. Their further replication and dissemination enhance potential human exposure, particularly for those working in agriculture or living near livestock farms.

**Figure 1:**
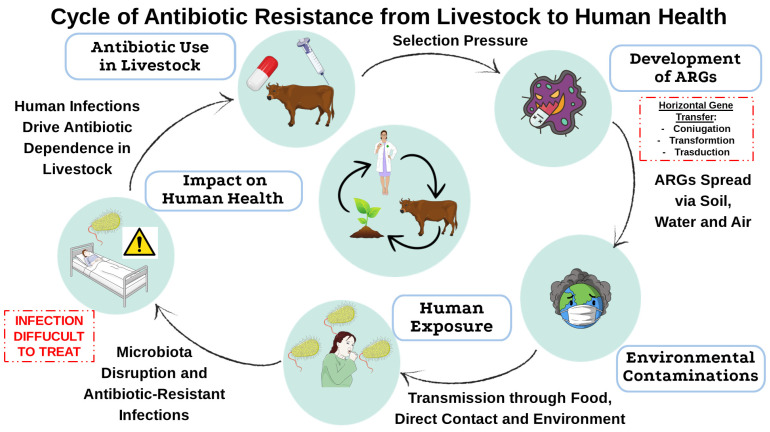
Transmission of antibiotic resistance from livestock to humans. This diagram illustrates how antibiotic use in livestock contributes to the selection of resistant bacteria, the spread of ARGs into the environment, and their eventual transmission to humans through food, direct contact, and environmental exposure. This cycle highlights the interconnected nature of antimicrobial resistance and its implications for public health. This image was created using the image bank of Servier Medical Art (Available online: http://smart.servier.com/; accessed on 30 April 2025) licensed under a Creative Commons Attribution 3.0 Unported License (available online: https://creativecommons.org/licenses/by/3.0/, accessed on 30 April 2025). ARGs: Antibiotic resistance genes.

Figure 1 illustrates the entire process of using antibiotics in livestock, from the generation and transmission of ARGs from soil, water, and air up to human exposure. These resistant bacteria can be introduced into humans in the consumed meals, via direct exposure to animals, or within the environment.

### 2.1. Mechanisms of Resistance Selection in Animals

Antibiotic resistance in livestock mainly arises through two mechanisms: vertical transmission and horizontal gene transfer. In vertical transmission, resistance emerges from spontaneous mutations in bacterial DNA and is passed down to daughter cells during replication [22]. De novo mutations can confer specific resistance to a specific antibiotic or class of antibiotics. Mutations in genes involved in DNA replication can confer resistance to fluoroquinolones [23]. However, in many cases, de novo mutations generate resistance to multiple unrelated antibiotics, leading to multidrug resistance (MDR) [24]. Mutations affecting efflux systems or membrane permeability contribute to multidrug resistance [25,26].

In contrast, horizontal gene transfer allows bacteria to acquire resistance genes from other microorganisms through processes such as conjugation, transformation, and transduction [22]. Conjugation has been identified as the most efficient route of ARG dissemination in livestock environments [27].

Horizontal gene transfer accelerates the spread of ARGs within bacterial populations [28]. Therefore, plasmids via the conjugative pilus allow the transfer of resistance genes between bacteria. Bacteria such as *Escherichia coli* transfer extended-spectrum beta-lactam (ESBL) plasmids to other strains, thereby conferring resistance to beta-lactams [29]. Mobile plasmids often carry genes like CTX-M-type β-lactamases, facilitating resistance dissemination [30]. In livestock, plasmids play a critical role in the horizontal transmission of AMR genes. For instance, plasmids containing resistance genes such as beta-lactamases and tetracycline resistance have been extensively characterized in foodborne *Escherichia coli* isolates. These plasmids, often belonging to *IncI*, *IncF*, and *IncX* incompatibility groups, facilitate the spread of resistance between bacterial species, enhancing the persistence and mobility of resistance genes [31].

Transformation is a process that allows the acquisition of resistance genes from other species by taking free DNA from the surrounding environment. *Streptococcus pneumoniae* is an example of a naturally competent human pathogen that can acquire resistance genes from resistant strains through the transformation process, allowing the emergence of new resistant strains [32]. In vitro transformation experiments in a laboratory strain have shown that the major determinants of β-lactam resistance are modified alleles of *pbp2x*, *pbp2b*, *pbp1a*, and *murM* [33]. Mutations in these alleles have also been associated as a cause of resistance for the first-line antibiotic amoxicillin [34].

Another mode of horizontal gene transfer is transduction, the process in which a bacteriophage particle transfers nonviral DNA from one bacterial host cell to another. In the human intestinal virome, it is estimated that the number of viruses is >10^12^ [35] and most of these viruses are bacteriophages (also known as phages). Therefore, they represent the most abundant gene transfer particles in the human intestinal microbiome [36]. Studies show that bacteriophages are abundant and carry a large amount of ARGs, making them important in horizontal gene transfer [37,38]. In fact, ARGs have been found in significant proportions in the DNA of bacteriophages from sewage, feces, seawater, and other environments [39]. Recent studies have shown that phages can transfer ARGs, including those for β-lactamases, between strains of *Pseudomonas aeruginosa*, contributing to the spread of antibiotic resistance, especially in livestock [40]. Some phages have been shown to transfer resistance genes at varying frequencies [41].

Antibiotic-resistant bacteria can acquire and transfer resistance genes from other sources, such as other plasmids or the bacterial chromosome, through mechanisms such as transposition, integron-associated gene cassette integration, and homologous recombination. These processes make resistance genes highly mobile, increasing their spread among bacterial species and in environments such as farms. Partridge et al. highlight how MGEs are associated with the spread of antimicrobial resistance and their ability to exchange resistance genes between bacteria [42]. This promotes the spread of antibiotic-resistant strains in the livestock environment. Transposons owe their intracellular mobility to the presence of insertion sequences (IS) from the same family acting together to move DNA between them, and they are important in the spread of antibiotic resistance in *Enterococcus faecalis* and *Enterococcus faecium* [43]. Insertion sequences can enhance resistance gene expression and promote virulence [44]. In addition, MGRs of enterococci, such as Inc18 plasmids, have been shown to promote the spread of resistance genes to more pathogenic bacteria such as *Staphylococcus aureus* [45].

Integrons are bacterial genetic elements that rearrange and express mobile gene cassettes, contributing to the spread of ARGs among pathogens [46]. For example, class 1 integrons confer resistance to aminoglycosides in Enterobacteriaceae isolates from several Chilean hospitals [47]. High levels of class 1 integron genes (intI1) have been detected in agricultural soils receiving livestock manure, as shown by Qian et al. [48], who reported significantly greater integron abundance in fertilized versus unfertilized soils, indicating their role in promoting ARG dissemination in farming environments [48].

Many of these mechanisms mentioned above may contribute over the long term to the persistence of resistance in environmental bacterial populations, including those on intensive livestock farms. The clinical rise in these resistance genes seems to be a result of the selective pressure exerted by antibiotics on bacteria that are either permanently or temporarily part of the human or domestic animal microbiome [49]. This dynamic underscores how the use of antibiotics on farms, although initially intended to control infections and improve productivity, may have long-term consequences for human health.

The key mechanisms described in this section are effectively summarized in Table 1, which provides a highly relevant overview of the genetic processes underlying antimicrobial resistance in livestock, including the types of gene transfer involved, the molecular elements responsible (e.g., plasmids, integrons, transposons), and representative examples of their impact.

### 2.2. Transmission Pathways of Antimicrobial Resistance to Humans

The increasing spread of AMR in zoonotic pathogens affecting both humans and animals represents a major public health concern [22], as emphasized by the World Health Organization (WHO) [50]. Likewise, the uncontrolled use of antibiotics in animal farming contributes to the emergence and dissemination of antibiotic-resistant bacteria [51]. The environment receives the largest quantity of antibiotic residue through waste and leakage from the pharmaceutical industry. Moreover, antibiotics administered to humans and animals are not fully metabolized, so they can be found in hospital and farm wastewater. These wastewaters also carry resistant bacteria and ARGs, facilitating their environmental dissemination [17,52]. The emergence of *Candida auris*, a multidrug-resistant fungus causing hospital outbreaks, highlights the urgency of implementing environmental AMR policies. Although its origin remains unclear, its presence in both hospitals and wastewater suggests a possible environmental reservoir [53]. Just such another example is *Capnocytophaga* spp., a bacteria often present in the oral cavities of animals. Although often benign, *Capnocytophaga* species have sometimes been associated with serious infections in immunocompromised patients, especially when delivered by bites or scratches. Some strains have exhibited resistance to beta-lactam antibiotics due to the production of beta-lactamases such as *Cfx*A (*Cephalosporinase gene A*), *CepA* (*Cephalosporinase of Bacteroides*), and *CblA* (*Cephalosporinase of Bacteroides Lineage A*), raising concerns about the role of companion animals as AMR reservoirs [54]. The ongoing overuse of antibiotics promotes the selection of resistant bacteria, which enhances their spread and increases the likelihood of human infection [55]. These resistant bacteria can be transmitted to humans through multiple routes, including food consumption, environmental exposure, and direct contact with farm animals or workers [56]. AMR transmission routes are often complex and difficult to trace or prevent. The two main pathways are (1) direct contact with food-producing animals, and (2) indirect acquisition through the food chain or polluted environments such as hospitals, manure, wastewater, and farmland [22].

It has been widely demonstrated that among individuals who have direct contact with animals, such as agricultural workers [57,58] and veterinarians [59] there is a high prevalence of antimicrobial-resistant bacteria. A research group has demonstrated a direct transmission of multi-drug-resistant *E. coli* from animals to animals and also from animals to humans, highlighting the importance of zoonotic transmission in acquiring antimicrobial-resistant bacteria. Antibiotics used in agriculture and human and animal medicine reach the environment in their active forms, and this causes a selective pressure that can lead to the emergence of antibiotic resistance phenotypes among various microbial species naturally occupying this niche [60]. The spread of resistance is regulated by numerous genes, many of which are highly transferable between bacterial species [55].

For example, the bla CTX-M genes originated from the environmental Gram-negative bacteria *Kluyvera* spp. [61]. Other examples that have already generated serious concerns worldwide due to their multi-drug-resistant phenotype are the carbapenem-resistant Enterobacteriaceae expressing enzymes such as KPC-2 (*Klebsiella pneumoniae* carbapenemase-2) and NDM-1 (New Delhi metal-β-lactamase-1) [62]. The enzyme NDM-1 confers resistance to almost all beta-lactams, and it is capable of spreading worldwide, raising concern about zoonotic transmission to humans [16].

Another well-documented demonstration of this phenomenon is the spread of resistance to colistin, first reported in 2016. This is mediated by plasmids carrying the *mcr-1* (Mobilized colistin resistance-1) gene among several species of the Enterobacteriaceae family [63]. This antibiotic has not only been used in clinical practice but is also widely used in veterinary practice and in animal feed to promote growth [64], significantly contributing to the emergence of colistin resistance [63].

These antibiotic-resistant bacteria and genes can spread in the environment not only through common routes but also through bioaerosols. ARGs present in livestock aerosols can enter the human body through upper airways, causing health risks [65]. Recently, bioaerosols have been recognized as a key mechanism for the spread of ARGs in pig farms [66]. Additionally, significant antibiotic-resistant airborne pathogen strains have been documented in agricultural animals’ farms. It has been confirmed by some studies that there is a connection between antibiotic resistance in the farm animals and their handlers such as farmers. A research team has examined the presence of methicillin-resistant *Staphylococcus aureus* (MRSA) in the air in four pig flocks, detecting the majority of MRSA and *S. aureus* in particles that can be deposited in the human upper respiratory tract, in primary, secondary, and terminal bronchi and alveoli [67]. Among multidrug-resistant bacteria, MRSA remains one of the most significant threats in both human and veterinary medicine. Initially associated with severe hospital-acquired infections, MRSA is now a major concern in livestock production [58,67]

The third route involves the direct transmission of obligate pathogenic bacteria via contaminated food; in some cases, contact with animals can also facilitate transmission, particularly leading to gastrointestinal infections (e.g., specific Salmonella serotypes) [68].

These disruptions of microbial ecosystems may be not readily detectable through standard clinical symptoms, but they have concrete consequences. The proliferation of antibiotic resistant bacteria in animal systems, and the transfer of those bacteria to people, especially among those working closely with livestock, is emerging as a growing public health concern [52].

## 3. Antibiotic Resistance and Public Health: The Role of Intensive Agriculture

Recent studies have confirmed that the improper use of antibiotics in livestock farming is a key factor in the enrichment and dissemination of ARGs in the environment. Manure and livestock wastewater, often containing unmetabolized antibiotics, facilitate the horizontal transfer of ARGs into soil, plants, and water systems, even after conventional treatment processes [69,70]. The potential risk of ARG transmission from livestock environments to human pathogens and its implications for treatment efficacy have been extensively reviewed in the literature [71]. The increase in infections caused by multi-resistant pathogens involves the use of prolonged and inefficient treatments, thus increasing healthcare costs and the risk of mortality, especially in frail individuals.

### 3.1. Environmental Dissemination of Resistance Genes from Intensive Farming

Antibiotics are widely and routinely used in livestock farms primarily to prevent diseases allowed to develop in intensive farming environments, and to a lesser extent as growth enhancers for sub-therapeutic concentrations that improve feed efficiency. Overuse of antibiotics (at sub-lethal concentrations) has led to the presence of antibiotics (albeit at low concentrations) in the animal tissue and gut of treated animals and in the environment [72]. In animals, antibiotics are known to stimulate intestinal vitamin synthesis, reduce intestinal bacterial flora, and alter rumen microbial metabolism. Furthermore, prolonged exposure to antibiotics exerts a selective pressure that can cause ARGs to develop in the gut microbiota of animals. Thus, livestock and poultry manure becomes a critical reservoir for ARGs and antibiotics. Qian et al. [48] detected 149 ARG subtypes in soils treated with livestock manure, with significantly higher diversity compared to unfertilized soil. Tetracycline-, sulfonamide-, and aminoglycoside-resistance genes were particularly abundant and were often associated with mobile genetic elements. Similarly, Zhang et al. [73] reported that swine and poultry manure, especially when spiked with antibiotics, significantly increased the abundance of ARGs in soil microcosms over time. These findings confirm that manure not only introduces ARGs but also creates conditions for their persistence and potential horizontal transfer [48,73]. According to FAO and other global assessments, between 75 and 90% of antibiotics used in livestock may be excreted unmetabolized, contributing significantly to environmental ARG contamination [74,75]. As reviewed by Li et al. [71], the use of manure in agriculture contributes to the environmental spread of ARGs, particularly through soil and plant systems linked to food production. Animal manure also contains hazardous compounds such as heavy metals, residual antibiotics, ARGs, and pathogenic bacteria. These contaminants can persist in the soil and, as demonstrated in experimental studies, may be taken up by plants or alter the plant-associated microbiota. As reported in experimental studies and comprehensive reviews [71,76], the application of manure as fertilizer poses a risk to human and animal health by facilitating the spread of ARGs into arable land and edible crops. Once penetrated into the soil microbiome, ARGs can be absorbed by plant-associated bacteria or contaminate water sources used for irrigation and consumption, thereby facilitating the transfer of resistance elements into the human food chain. Furthermore, intensive agriculture generally uses commercially formulated feeds, whereas traditional systems use a more diverse array of feed types, including silage, forage grasses, legumes, cereals, and others. As a result, the concentrations of heavy metals, antibiotics, and ARGs in livestock and poultry feces can vary significantly depending on the farming system used [77]. One study reported that ARG levels in manure-treated farmland were 100 times higher than those in unfertilized soil [78]. Another study found that farmland treated with chicken manure for 10 consecutive years exhibited a significant increase in both the abundance and diversity of ARGs, with these resistance genes also accumulating in plants [79]. Moreover, even when manure is processed into commercial organic fertilizer and applied to soil, it still contributes to the enrichment of both the diversity and abundance of ARGs and MGEs in the environment.

In food-producing animals, the use of antibiotics promotes the transfer of ARGs between enteric bacteria in the intestinal tract, thereby inducing antibiotic resistance in the fecal microbiome [80]. In some countries, the use of antibiotics for medical purposes only has been largely restricted; however, there are still many countries with high zootenic production that still misuse them. Eighteen classes of antimicrobials have been approved by the Food and Drug Administration (FDA) for use in food-producing animals [71]. Among the most frequently detected antibiotics in livestock manure are tetracyclines (such as oxytetracycline and chlortetracycline), tylosin, sulfamethazine, monensin, virginiamycin, and penicillin. Recent analytical studies have confirmed their widespread presence in manure from swine, poultry, and cattle farms, with tetracyclines showing particularly high concentrations [78]. Thus, the widespread use of these molecules in livestock practices not only favors their environmental dispersion through manure, but also contributes to the selection and accumulation of specific resistance genes in the gut microbiota of animals such as *tetM*, *tetW*, and *tetQ* in the gut microbiota of cattle [81].

In conclusion, the intensive use of antibiotics in animal husbandry is progressively reducing the efficacy of available antibiotics, both in human and veterinary medicine, increasing the risk of difficult-to-treat diseases and driving up costs for the livestock sector.

The relationship between the environment, animals, and human health makes it increasingly clear that an integrated approach, known as “One Health”, is needed to tackle the problem of antibiotic resistance in an effective and coordinated manner.

In addition to limiting the use of antibiotics, targeted agricultural interventions and health policies are needed to limit the spread of resistance between animals, the environment, and humans.

### 3.2. Antibiotic Resistance in Livestock and Its Effects on Animals and Human Gut Microbiota

The overuse of antibiotics in livestock farming has consequences for both the animals themselves and also for the human microbiome. When livestock are treated with antibiotics, resistant bacteria and ARGs can be released into the environment through animal waste. It has recently been shown that environments harbor microbial communities that can act as hotspots for enrichment and exchange of ARGs.

The presence of resistant bacteria in the environment contributes to the destruction of microbial ecosystems, including the gut microbiota of livestock. Antibiotics, by selecting resistant bacteria, can alter the composition of the microbiome in animals, causing dysbiosis [82]. The gut microbiota plays an important role in human health by influencing numerous vital processes, including defense against pathogens, immune system development, and regulation of drug availability and pharmacokinetics [83,84]. Disruption of the gut microbiota in both livestock and humans due to antibiotics can lead to multiple health issues, increasing vulnerability to pathogenic bacteria and changes in metabolic pathways. Moreover, gut microbiota alterations are often invisible, but have important consequences for both animal and human health. Dysbiosis, often asymptomatic, refers to an imbalance in the microbial community associated with disease states and may result from diet, antibiotic use, stress, or pathology [85]. A balanced gut microbiota, due to the hypoxic nature of the gut, is more populated by obligate anaerobes and plays a key role in maintaining homeostasis, energy metabolism, and the supply of essential biomolecules that are not synthesized by the host [86]. In calves, preventive antibiotic treatment has been shown to cause gut dysbiosis, characterized by a significant expansion of facultative anaerobic *Escherichia coli*, a key indicator of microbial imbalance [87]. Facultative anaerobic bacteria such as *E. coli* thrive in oxygenated environments, promoting aerobic respiration, increased gut permeability, and altered microbial balance [88]. Differences in gut microbiota composition between obese and lean hosts have been documented in both experimental animal models and clinical human studies, highlighting its involvement in energy homeostasis and fat accumulation [89,90].

Gut dysbiosis has been strongly associated with the pathogenesis of inflammatory bowel diseases (IBD). In particular, dysbiosis alters bile acid metabolism, leading to a reduction in anti-inflammatory secondary bile acids and an increase in their sulphated forms, which can enhance the inflammatory response in the intestinal epithelium [91]. The impact of antibiotics on the bacterial community may also contribute to the development of colorectal cancer, as suggested by experimental studies in animal models. This supports the idea that gut microbiota composition plays an important role even in the onset of intestinal tumors [92].

In animals, dysbiosis can lead to reduced welfare, slower growth, and increased susceptibility to various diseases, including gastrointestinal disorders and metabolic diseases [93]. Such alterations can promote small intestinal bacterial overgrowth SIBO and increased intestinal permeability, commonly referred to as leaky gut syndrome [94]. For example, dysbiosis-induced dysbiosis in mice has been shown to impair overall health and reduce reproductive and adaptive fitness, particularly affecting female-to-male mating behavior. These findings demonstrate that the gut microbiota influences not only reproductive behavior [95], but social behavior, general health, and overall fitness of the host [96,97].

In conclusion, the disruption of the intestinal microbiota caused by antibiotic treatments can further promote the growth of both commensal and pathogenic bacteria carrying a wide range of MDR. These bacteria, equipped with highly mobile plasmids, can transfer resistance genes between agricultural and clinical environments. This phenomenon, which has been largely overlooked in the past, underscores the urgent need to address the consequences of widespread antibiotic use in both agricultural and clinical settings [88]. However, the etiology between the propagation of ARGs from animal farming and the acquisition of ARGs by the human gut microbiome is still unclear [52].

**Figure 2:**
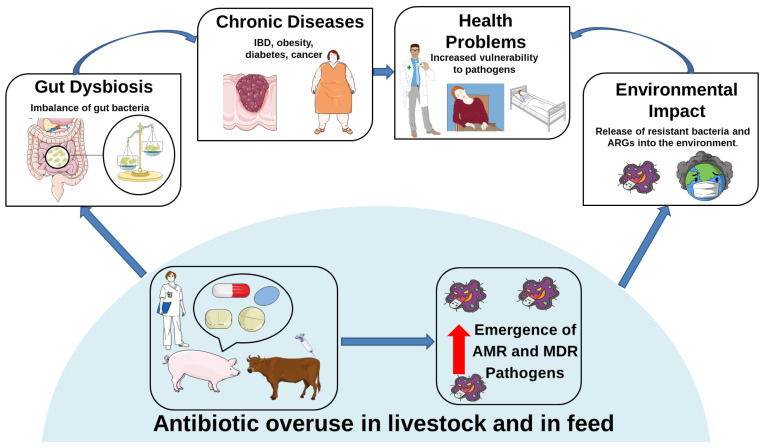
Antibiotic overuse in livestock and in feed. The overuse of antibiotics in animal husbandry promotes the emergence of MDR pathogens, contributing to gut dysbiosis, increased health problems, the onset of chronic diseases, and the environmental spread of ARGs and MDR pathogens. The red upward arrow indicates the increase in AMR and MDR pathogens as a consequence of antibiotic overuse. This image was created using the image bank of Servier Medical Art (Available online: http://smart.servier.com/; accessed on 30 April 2025) licensed under a Creative Commons Attribution 3.0 Unported License (available online: https://creativecommons.org/licenses/by/3.0/, accessed on 30 April 2025). ARGs: Antibiotic Resistance Genes; MDR: Multidrug Resistance.

Figure 2 illustrates how excessive antibiotic use in livestock triggers a cascade of adverse effects, from MDR emergence to environmental contamination, gut dysbiosis, and human health risks.

## 4. Antibiotic Use in Livestock as a Hidden Driver of Neuroinflammatory Risk

**Figure 3:**
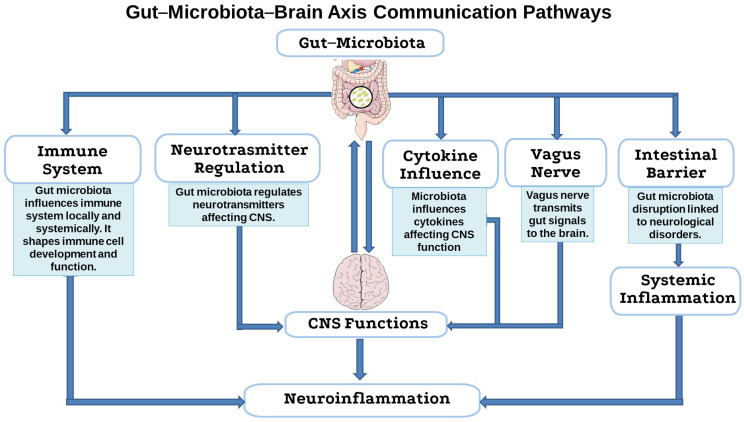
Flow chart of the gut-microbiota–brain axis communication pathways. This flow chart illustrates the main pathways through which the gut microbiota communicates with and influences central nervous system (CNS) functions. These interactions occur via the immune system, neurotransmitter regulation, cytokine signaling, vagus nerve transmission, and the integrity of the intestinal barrier. These mechanisms collectively contribute to systemic inflammation and neuroinflammation, with potential implications for neurological and psychiatric disorders. This image was created using the image bank of Servier Medical Art (Available online: http://smart.servier.com/; accessed on 1 June 2025) licensed under a Creative Commons Attribution 3.0 Unported License (available online: https://creativecommons.org/licenses/by/3.0/, accessed on 1 June 2025). CNS: central nervous system.

The gut-microbiota–brain axis is a bidirectional communication network linking the gastrointestinal tract and the central nervous system (CNS) [98]. Despite the anatomical separation, there are several pathways through which the gut microbiota communicates with the CNS. These include modulation of the enteric nervous system (ENS), the immune system, the neuroendocrine system, and the circulatory system through the production of neuroactive substances, hormones, and metabolites (Figure 3) [99]. It is well known that the gut microbiota influences the immune system both locally in the gut and systemically. Alterations in peripheral immunity can, in turn, affect the CNS, contributing to neuroinflammation and neurodegeneration [99]. A crucial element in this pathway is the intestinal barrier, which regulates the passage of substances from the intestinal lumen into the bloodstream. Microbiota-derived metabolites, such as short-chain fatty acids (SCFAs), secondary bile acids, and amino acid metabolites, can strengthen the integrity of the intestinal barrier or, in cases of dysbiosis, lead to increased intestinal permeability (“leaky gut”), facilitating the translocation of pro-inflammatory factors, bacterial products (such as lipopolysaccharide, LPS), and even bacteria themselves into systemic circulation [100]. The microbiota also influences the development and function of immune cells, such as regulatory T cells, T helper 17 cells, and mucosal-associated invariant T cells. These cells, shaped by the gut microbiota, can migrate to the CNS and contribute to neuroinflammation [101]. Additionally, the microbiota, often via SCFAs, provides essential signals for the maturation and activation of microglia, a type of CNS-resident glial cell that serve as the brain’s primary immune cells and critical mediators of the gut-microbiota–brain axis. The microbiota also influences cytokines, immune signaling molecules, that cross the blood–brain barrier (BBB) and can affect the CNS. For example, IL-17 and IL-6 have been linked to neurodevelopmental disorders [102]. The bidirectional gut–brain neuronal communication pathways primarily involve the ENS and the vagus nerve. Microbial-derived metabolites can regulate enteric neuronal functions, such as the excitability of enteric nerve endings, indirectly influencing endocrine and immune pathways, and thereby interacting with the CNS [103]. The vagus nerve plays a crucial role in gut–brain communication. Its sensory (afferent) fibers transmit signals from the gut to the brain, while motor (efferent) fibers send signals from the brain to the gut. Vagal afferents detect chemical and mechanical signals from the gut [104]. Additionally, enteroendocrine cells release neurohormones such as glucagon-like peptide 1, peptide YY, cholecysto-kinin, substance P, and serotonin in response to microbial signals [105]. These hormones bind to receptors on vagal sensory neurons, transmitting information to the CNS. It seems that the gut microbiota interacts indirectly with mechanosensors to regulate neurodevelopment. A special subtype of enteroendocrine cells, known as neuropod cells, synapse with vagal neurons, enabling rapid signal transmission from the gut to the brain, often using glutamate as a neurotransmitter [104,106]. The vagus nerve is essential for peptides or neurotransmitters derived from microbial metabolites or host secretions (such as serotonin, oxytocin, γ-aminobutyric acid GABA, brain-derived neurotrophic factor) to exert their effects on the brain. For instance, the vagus nerve mediates the effect of *Lactobacillus rhamnosus* on regulating GABA receptors in the brain [102]. The gut microbiota, by stimulating neurotransmitters such as γ-aminobutyric acid GABA [107], serotonin [108], and dopamine [109], is capable of regulating CNS functions. Gut bacteria are involved in the biosynthesis of serotonin and regulate its concentration by influencing the CNS [110]. Dopamine is also regulated by the gut microbiota, affecting dopaminergic signaling and the progression of diseases such as Parkinson’s disease (PD) [111]. The gut microbiota can influence GABA levels and receptors, as well as cholinergic signaling, which is important for cognitive function [112]. Glutamate, although it does not readily cross the BBB, is influenced by its precursor glutamine, which is also affected by the microbiota and probiotics [113].

Thanks to recent technological advances, researchers have begun to uncover the complex ways in which the microbiome communicates with the rest of the body. Both preclinical and clinical studies have shown that when the balance of gut microbes is disrupted, it may play a role in the development of conditions such as Autism Spectrum Disorders [114], anxiety [103], depression [115], reduced motivation [116], and even neurodegenerative diseases [117,118,119]. Emerging evidence also suggests a possible link between gut microbiota alterations and attention-deficit/hyperactivity disorder (ADHD). Dysbiosis may influence neurotransmitter levels, immune activation, and brain function, contributing to ADHD-related symptoms [120]. Antibiotics, especially when used indiscriminately in livestock, can reach humans via the food chain or environmental exposure, potentially disrupting the gut microbial balance [121,122]. Such disruptions are associated with impaired gut-microbiota–brain axis signaling and can contribute to increased intestinal permeability and systemic inflammation. This systemic immune activation can cross the BBB, promoting neuroinflammation, a critical factor implicated in the pathogenesis of neurodegenerative diseases [123,124]. Therefore, the gut-microbiota–brain axis represents a potential therapeutic target for these diseases.

### 4.1. Microbial Balance and the Integrity of the Gut-Microbiota–Brain Axis

The imbalance of the gut microbiota promotes various pathological conditions and affects developmental and metabolic processes in extra-intestinal organs, including the brain. In these organs, the microbiota is involved in the production of metabolites and neurochemicals; conversely, neurotransmitters such as adrenaline and noradrenaline from the host influence the growth and virulence of bacteria [125]. This bidirectional communication is essential for regulating neurotransmitter production, which underlies cognitive and emotional functions. The gut-microbiota–brain axis contributes to brain development, myelination, neurogenesis, pain perception (nociception), and hypothalamic–pituitary axis activity [126]. This two-way communication between the microbiota and the central nervous system takes place mainly through the parasympathetic nervous system, involving autonomous neurological, hormonal, and immune regulatory processes via mediators such as cytokines and chemokines [127]. In addition to cytokines and chemokines, neuroendocrine peptides such as glucagon-like peptide-1, peptide YY, and cholecystokinin, released by enteroendocrine cells in response to microbial signals, play a key role in this communication. These molecules interact with vagal afferent fibers and can influence brain function, including neuroinflammation, appetite regulation, and even cognitive processes. Glucagon-like peptide-1, in particular, has been investigated for its potential neuroprotective effects and its ability to modulate neuroimmune pathways [128]. Disruption of this balance (e.g., through antibiotic exposure) can impair gut–brain communication. Notably, this axis includes not only microorganisms but also their genetic material and metabolic products. Animal studies involving antibiotics, probiotics, microbiota transplants, or germ-free models have confirmed this communication [129]. One key study by Diaz Heijtz et al. [130] showed how the “normal” gut microbiota influences brain development and behavior in mammals, using germ-free (i.e., microbiota-free) and specific pathogen-free (with normal microbiota) mouse models. The germ-free mice showed behavioral changes compared to the experimental group with gut microbiota, and exhibited reduced anxiety, increased motor activity, and increased risk-taking behavior. Related to this, the germ-free animals observed an increased turnover of noradrenaline, dopamine, and serotonin in the striatum, a brain region linked to motor control and anxiety. These animals also showed low expression of key synaptic plasticity genes (nerve growth factor-inducible clone A and brain-derived neurotrophic factor) in brain regions such as the prefrontal cortex, striatum, hippocampus, and amygdala, and higher expression of synaptophysin and PSD-95 in the striatum. These results reinforce the hypothesis that dysbiosis induced, for example, by antibiotic use, may have lasting effects on neurodevelopment [130]. In compliance with these data, another group of researchers demonstrated that the absence of microbiota during the early stages of life alters neuronal activity. Indeed, in germ-free mice, over-regulation of immediate response genes was observed that altered neuronal activity and induced hyperexcitability of the amygdala [131].

Research on the gut microbiome and the gut-microbiota–brain axis in farm animals is still limited, mainly due to practical difficulties such as the need to breed germ-free animals in specialized and cumbersome facilities. However, studies on the effects of probiotics provide further evidence of the role of the microbiota in modulating the behavior and cognitive abilities of animals, with consequences for their health. The study conducted by Val-Laillet et al. examined the impact of a Western-style diet given to mothers during gestation and lactation, evaluating the consequences on puppies. Maternal diet influenced not only the mother’s but also the puppies’ microbiota, even regulating cognitive responses and hippocampal neurogenesis in puppies [132].

### 4.2. Antibiotic-Driven Dysbiosis as a Trigger for Neuroimmune Activation

Recent experimental evidence continues to reveal how dysbiosis-driven inflammation affects neuroimmune function, particularly in relation to antibiotic use in livestock. The gut microbiota, a complex community of microorganisms, is essential for normal neurodevelopment, immune maturation, and protection from pathogens in humans and animals. Maintaining a healthy gut microbiota is not only about gut health, but is also essential for protecting the brain. This bidirectional communication suggests a potential role for gut microbiota-targeted interventions in influencing neurological outcomes, although further studies are needed to clarify their therapeutic applicability in neurodegenerative conditions [133].

Animal studies show that antibiotic-induced microbiota depletion can increase levels of LPS, a bacterial endotoxin, leading to neuroinflammation and behavioral changes [134]. LPS, by damaging the intestinal epithelial barrier, increases its permeability (“leaky gut”), allowing toxins and metabolites to enter the bloodstream [13]. This triggers a chronic systemic inflammatory response. In addition to systemic effects, dysbiosis can also cause localized intestinal inflammation. This involves activation of enteric glial cells and peripheral neurons within ENS, which relay inflammatory signals to the brain through neuroimmune pathways [135]. This immune activation can lead to the recruitment of central immune cells, such as microglia and astrocytes, in the CNS, amplifying neuroinflammatory responses initiated at the gut level [136]. In addition, LPS can interact with proteins such as α-synuclein and beta-amyloid (Aβ), promoting their aggregation [137]. Some studies suggest that altered gut microbiota may influence the synthesis of neuroactive compounds, potentially affecting neurodevelopment and behavior, with observed associations to symptoms such as anxiety, depression, and cognitive changes relevant to ASD models [138,139].

Some studies show that the use of probiotics (*Bacillus subtilis*, *Lactobacillus rhamnosus*) reduces stress-induced harmful behaviors in laying hens, acting on the restoration of the gut microbiota and on serotonin metabolism in hens with antibiotic-induced dysbiosis [140,141]. This suggests a link between antibiotic-induced dysbiosis and the impact on the gut-microbiota–brain axis, including neuroinflammation and behavior.

Microbial products such as LPS can interact with proteins such as α-synuclein and Aβ, promoting their aggregation. In addition, some bacteria produce proteins that participate in the accumulation of neurofibrillary plaques and tangles [9,10]. For instance, the secretome of *Akkermansia muciniphila* has been shown to promote α-synuclein aggregation in enteroendocrine cells [142]. In contrast, beneficial metabolites such as short-chain fatty acids (SCFAs) produced by commensal bacteria can interfere with protein aggregation. Bacteria such as *Bacteroides ovatus* produce phenolic acids that hinder α-synuclein aggregation [143], while *Bacillus subtilis* can inhibit aggregation by modulating metabolic pathways such as sphingolipids [144]. The accumulation of misfolded proteins (α-synuclein, Aβ, tau) is a characteristic feature of neurodegenerative diseases, often caused by a dysfunction of clearance systems such as the autophagy–lysosome and proteasome [145]. Thus, chronic inflammation induced by dysbiosis can weaken these functions. In a mouse model of AD, the germ-free state reduced brain Aβ load compared to conventionally bred mice, suggesting that antibiotic-induced microbiota alterations may influence neuroinflammation and amyloidosis [146]. In contrast, in another mouse model of AD (5XFAD model), antibiotic administration reduced activation of the C/EBP-β/AEP pathway in the brain, decreased pro-inflammatory microglia signals, reduced aggregation of Aβ fibrils, and improved cognitive function. These findings indicate that, under specific experimental conditions, modulating the microbiota through antibiotic treatment may confer neuroprotective effects, possibly by reducing inflammatory mediators or microbial metabolites, but this remains to be further validated [147]. In a genetically modified PD mouse model (Pink1-KO), an intestinal infection caused by a pathogenic bacterium (*Citrobacter rodentium*) triggered immune activation and the recruitment of mitochondria-specific CD8^+^ T cells in the brain. This induced dopaminergic neuronal dysfunction and motor deficits. After infection, no substantial change in microbial diversity was determined, although an immune response varied in Pink1-KO mice, and a disproportionate increase in butyric acid (an SCFAs) was observed in these mice, possibly as a compensatory response [148]. This example illustrates how microbial disturbances, such as those caused by infections, may influence neuroinflammatory and immune pathways in susceptible models, potentially resembling features seen in neurodegenerative diseases like PD. Furthermore, this study highlights the role of gut dysbiosis in triggering autoimmune responses. Indeed, the idea of molecular mimicry has been suggested, where bacterial structures resemble host proteins, leading the immune system to attack their own tissues, causing autoimmune reactions.

Thus, microbial metabolites and systemic inflammation can compromise the integrity of the BBB, which allows pro-inflammatory molecules, bacterial toxins, and peripheral immune cells to infiltrate the CNS, thereby fueling neuroinflammation and neuronal damage [149].

Moreover, dysbiosis alters the host metabolomic profile by reducing beneficial SCFAs, such as acetate, propionate, and butyrate, which are essential for maintaining intestinal barrier function and exert anti-inflammatory and neuroprotective effects [150]. Conversely, it increases harmful metabolites including LPS, beta-methylamino-L-alanine, and secondary bile acids, which have been associated with cognitive impairment and may negatively affect BBB and brain metabolism [151,152].

In conclusion, the use of antibiotics in livestock farming, as antibacterial agents, can significantly disrupt the composition of the gut microbiota in animals. This leads to a reduction in microbial diversity and the induction of dysbiosis, with a negative impact on animal health. Antibiotic-induced dysbiosis has been linked to elevated levels of LPS in animal models, a bacterial endotoxin capable of triggering systemic inflammatory responses. This is where the crucial connection with neuroinflammation and behavioral changes in animals emerges. Taken together, the evidence presented, summarized in Table 2, suggests that extensive antibiotic use in livestock may alter gut microbial composition in ways that have implications for systemic inflammation and, potentially, neurological health. However, more studies are needed to firmly establish causal links in this complex axis. It is worth noting that not all studies support a direct link between antibiotic-induced dysbiosis and neurodegeneration, and some models have shown neutral or even beneficial effects depending on context, dosage, and microbial composition. This variability highlights the need for further investigation before drawing firm conclusions.

**Figure 4:**
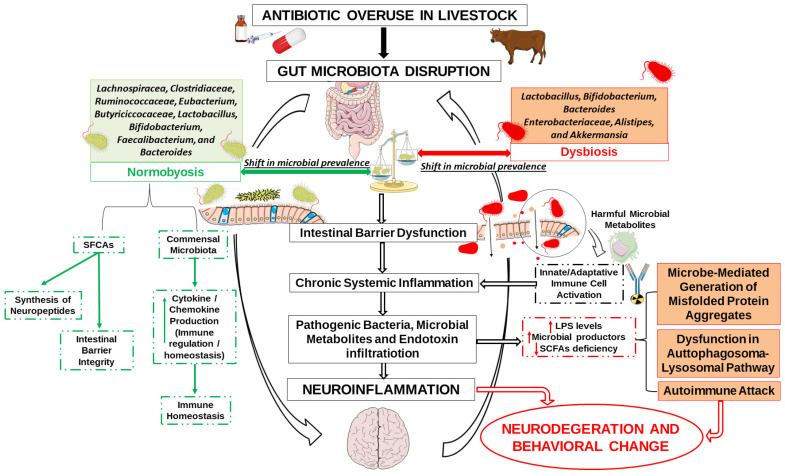
Pathways by which antibiotic overuse in livestock can affect neurological health through gut microbiota dysbiosis. The figure shows the pathogenic progression from antibiotic overuse in livestock to neuroinflammation and behavioral changes. Antibiotic resistance and dysbiosis lead to a decrease in beneficial bacteria and an increase in opportunistic strains, compromising the integrity of the intestinal barrier. This increases (Arrow pointing upwards) the access of microbial metabolites and LPS to the systemic circulation causing inflammation and immune activation. These processes contribute to neuroinflammation and are associated with misfolded protein aggregation, autophagosomal dysfunction, and autoimmunity, which can induce neurodegeneration and behavioral disorders. Reduced SCFA (Arrow pointing downwards) availability further exacerbates these effects by impairing intestinal barrier integrity and immune homeostasis. Note: The microbial changes depicted in the normobiosis and dysbiosis panels represent a shift in microbial prevalence, not absolute presence or absence. The cytokine/chemokine signals (Arrow pointing upwards) illustrated in the normobiosis pathway refer to immune-regulatory mechanisms supporting mucosal homeostasis. This image was created using the image bank of Servier Medical Art (Available online: http://smart.servier.com/; accessed on 30 April 2025) licensed under a Creative Commons Attribution 3.0 Unported License (available online: https://creativecommons.org/licenses/by/3.0/, accessed on 30 April 2025). SCFAs: short-chain fatty acids; LPS: lipopolysaccharides.

Maintaining a healthy gut microbiota is not only about gut health, but is also essential for protecting the brain. It is also important to note that most studies on gut microbiota rely on fecal samples, which primarily reflect luminal bacteria. This approach may overlook mucosa-associated microbial communities, which are in closer contact with the intestinal epithelium and play a critical role in modulating host immune and neural responses [153]. Including analyses of mucosal samples, when ethically and practically feasible, could offer a more comprehensive understanding of gut microbiota–host interactions, particularly in the context of neuroinflammation and gut-microbiota–brain axis research. These interactions are visually summarized in Figure 4, which illustrates how antibiotic-driven microbial alterations may influence neuroinflammatory outcomes.

## 5. Mitigation Strategies

### 5.1. Reducing Antibiotic Use in Livestock

Sustaining animal health and welfare is essential for achieving satisfactory levels of productivity and reproductive performance [154]. The implementation of antimicrobial stewardship programs in the healthcare sector and the revision of livestock management regulations are still progressing slowly, although it is well known that the overuse of antibiotics causes harmful effects. Practical examples of stewardship include maintaining detailed records of antibiotic use, enforcing withdrawal periods before slaughter, and requiring veterinary prescriptions for all treatments. We need to promote and adopt alternative strategies to antibiotic use for mitigating the rise of AMR in livestock. In this regard, international organizations such as the WHO, World Organisation for Animal Health, and the Food and Agriculture Organization of the United Nations have issued joint guidelines to reduce antimicrobial use in animals as part of the global “One Health” approach. Denmark has implemented successful measures including stricter antimicrobial use controls, a ban on antibiotics for growth promotion, and improved biosecurity [155]. Data from high-income countries show that strong surveillance systems are key to reducing antibiotic use in livestock.

Antibiotics are primarily used for therapeutic or preventive purposes in the cattle industry. Several antibiotic alternatives have been proposed, including prebiotics, probiotics, vaccines, synergistic drug combinations, antimicrobial peptides, and antimicrobial polymers [156]. Probiotics and prebiotics are promising alternatives and are discussed in greater detail in the following Section 5.2.

Vaccination also plays a crucial role, as it helps reduce the need for antibiotics by decreasing both the incidence and severity of infections. Significant progress has been made in developing vaccines to address common cattle health issues, including tick infestations, parasitic infections, respiratory diseases, and liver abscesses [157]. Many of these vaccines are still underdeveloped and require further testing. This is particularly important, as respiratory infections are one of the leading causes of antibiotic use in cattle farming [158]. An effective vaccine could help reduce this need.

Another promising strategy is combination therapy, which involves the use of two or more antibiotics, or the combination of antibiotics with non-antibiotic agents such as antimicrobial peptides, polymers, or immune response modulators [159]. This approach enhances treatments efficacy against resistant pathogens and reduces the likelihood of their survival and spread. A well-known example is the combination of β-lactam antibiotics with β-lactamase inhibitors. Despite these promising developments, practical and effective alternatives in pasture-based livestock systems remain limited [156]. Therefore, it is essential to improve veterinary infrastructure, promote preventive care, and invest in education for both farmers and veterinarians [147]. Raising awareness across the livestock sector from farm workers to producers can help shift behavior and reduce future risks of AMR [160,161].

Finally, stronger biosecurity measures are needed to reduce the risk of bacteria transmission between animals and from animals to humans. These measures should include reducing direct contact, enhancing hygiene practices, and strengthening sanitation in dairy systems [162].

### 5.2. Probiotics and Prebiotics

Nowadays, researchers increasingly support the use of probiotics to regulate the gastrointestinal microbiota and improve host health.

Probiotics are live microorganisms naturally present in the gastrointestinal tract. They produce metabolites that support the growth of beneficial bacteria, inhibit pathogenic bacteria, regulate pH, stimulate mucus production and enhance the function of intestinal epithelial cells [163]. In livestock production, probiotics are used to improve gut health, increase feed efficiency, and enhance milk quality. Their administration can also help prevent dysbiosis, especially under stress conditions such as transport. In animal models, probiotics have been shown to mitigate intestinal dysbiosis induced by deoxynivalenol, a common mycotoxin contaminating cereal-based food and feed [164,165]. For instance, *Lactobacillus murinus* alleviates deoxynivalenol-induced growth retardation and promotes an M2 anti-inflammatory response in the gut [166]. Other strains, *Saccharomyces cerevisiae*, *Lactobacillus*, and *Bifidobacterium*, have also demonstrated beneficial effects by modulating the gut microbiota composition and promoting host health [167].

Prebiotics, on the other hand, are substrates, such as non-starch polysaccharides or oligosaccharides, that are indigestible by the host but fermentable by the gastrointestinal microbiota, and promote the growth of beneficial bacteria [168]. Indeed, the use of prebiotics can promote weight gain, feed efficiency and general animal welfare. For example, fructooligosaccharides and galactosyl-lactose have been shown to reduce enteric disorders and enhance growth in calves [169]. These compounds are fermented in the large intestine by beneficial bacteria, improving gut health, microbial balance, and fecal quality [170]. Indeed, in livestock and poultry production, these prebiotics are widely used to regulate the gut microbiota, minimize fecal odors, and improve growth performance. In pigs, dietary supplementation with prebiotics has been shown to promote growth, strengthen both innate and adaptive immunity, and improve intestinal mucosal structure [171].

Although several controlled studies have demonstrated the effectiveness of probiotics and prebiotics in modulating the gastrointestinal microbiota, microbial dynamics and function outcomes remain incompletely understood. Further research is needed to better elucidate how these agents influence gut microbial balance and overall host health.

Given their potential role in maintaining microbial homeostasis, probiotics and prebiotics could serve as key components in future “One Health” strategies aimed at reducing antimicrobial resistance and improving both animal and human health.

### 5.3. “One Health” Approach

The “One Health” philosophy is considered fundamental to addressing the complex and multifaceted global crisis of antibiotic resistance, which poses a serious threat to livestock, human beings, and the environment. This interconnectedness of animal, human, and environmental health is the reason why antibiotic resistance is the paradigmatic example of the “One Health” strategy. Antibiotic-resistant bacteria and resistance are shared across these interconnected domains through well-established transmission pathways. The use of antimicrobials in any of these fields can potentially exacerbate the problem of AMR [172].

Due to the interplay between genetic adaptation and ecological interactions, resistant bacteria are now widely distributed across various environmental compartments—such as water, soil, and biological taxa—and are also present throughout the human food chain, including poultry, livestock, aquaculture, and crops [1].

The “One Health” approach is a collaborative, multidisciplinary strategy that operates at local, national, and global levels, aiming to achieve optimal health for humans, animals, plants, and the environment. Effective mitigation strategies under this framework require the coordinated involvement of diverse disciplines and relevant authorities across these levels [173]. The “One Health” philosophy is now an integral part of global efforts to combat antimicrobial resistance. The World Organisation for Animal Health, the Food and Agriculture Organization of the United Nations, and the WHO have launched the Global Action Plan on Antimicrobial Resistance to guide AMR policy within the “One Health” framework [174]. These organizations have also developed guidelines for monitoring AMR transmission and antimicrobial usage across animals, humans, and environmental systems. However, major challenges in addressing AMR arise from conflicting interests among different societal sectors affected by environmental, human, and animal health concerns. The effective implementation of these interventions though requiring time and strategic coordination is essential for preserving progress in global health and development [4]. In response to these challenges, stakeholders have identified key areas of intervention and optimal strategies to monitor antimicrobial resistance, contain infections, and inform antimicrobial stewardship policies.

In implementing the “One Health” approach to tackle AMR, several advances have been made. The “One Health” paradigm has become an integral part of global initiatives aimed at combating AMR. These organizations have introduced a Global Action Plan on AMR within the “One Health” framework and have established guidelines for integrated antimicrobial resistance surveillance to monitor the use and consumption of antimicrobials in the animal, human, and environmental sectors [1]. There is also a shared understanding regarding critical areas of action, AMR monitoring, infection containment, and policies governing antimicrobial use among various stakeholders.

However, several significant challenges remain. AMR is an inherently complex and multifaceted problem. A major challenge is managing the divergent interests of various social sectors and groups involved in animal, human, and environmental health. Moreover, there is still a lack of concrete explanations regarding the spread of AMR and the directions of its transmission [175]. Improving global surveillance, which is essential, requires a deeper understanding of antibiotic consumption in human and animal settings, current levels of AMR, and the molecular basis of resistance. Effective international collaboration between countries is also considered essential to address AMR through the “One Health” approach. Despite progress in recognition and planning, the complexity of the issue, conflicting sectoral interests, and the need for broader and deeper understanding and cooperation remain key challenges.

**Figure 5:**
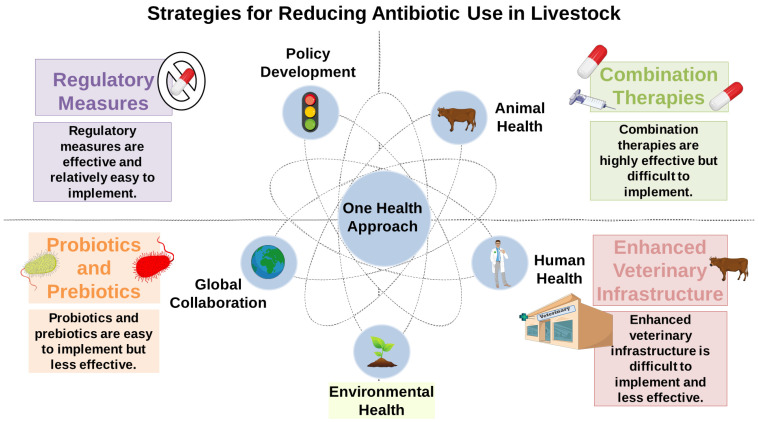
Strategies to decrease antibiotic use in livestock under the “One Health” framework. The chart depicts a holistic strategy that includes regulatory measures, combination therapy, probiotics/prebiotics, enhanced veterinary infrastructure, and global cooperation as complementary methods to reduce the use of antibiotics in agriculture. All interventions vary in efficiency and implementability, but all are for the good of animal, human, and environmental health according to the premises of the “One Health” paradigm. This image was created using the image bank of Servier Medical Art (Available online: http://smart.servier.com/; accessed on 30 April 2025) licensed under a Creative Commons Attribution 3.0 Unported License (available online: https://creativecommons.org/licenses/by/3.0/, accessed on 30 April 2025).

In light of the growing urgency surrounding antimicrobial resistance, there is a critical need to implement integrated measures aimed at reducing antibiotic dependency in livestock production. As shown in Figure 5, “One Health” has been advocating multifactorial measures ranging from policy regulation and health policy formulation to combination therapy, probiotics, and improved veterinary infrastructure. Achieving a balanced and interconnected relationship between animal, human, and environmental health is essential for sustainably addressing the global antimicrobial resistance crisis.

## 6. Conclusions and Future Perspectives

The excessive use of antibiotics in livestock farming has become a global public health emergency, contributing both to the spread of antimicrobial resistance and intestinal dysbiosis with potential neuroinflammatory effects. In this scenario, the “One Health” policy that considers human, animal, and environmental health as a single approach will be able to counteract the expansion of the global resistome and its neurobiological consequences.

To understand the molecular mechanisms through which antibiotic-induced dysbiosis contributes to neuroinflammation and neurodegeneration, it will be necessary to perform longitudinal studies, especially on the most exposed populations such as agricultural workers. These could be useful to identify early biomarkers responsible for dysbiosis and the consequent systemic immune response. Moreover, future research should explore the presence and bioactivity of antibiotic residues and resistant bacteria transmitted through the food chain or environmental exposure, particularly in vulnerable human populations.

To support microbial homeostasis and mitigate the neuroimmune risks associated with dysbiosis, future strategies should also focus on implementing stricter antibiotic stewardship in agriculture, promoting dietary interventions (e.g., fiber-rich or prebiotic-containing diets), and developing targeted probiotics or postbiotics capable of modulating gut–brain communication. These approaches may offer promising avenues to maintain or restore microbial diversity, which is essential for both neurological and systemic health.

In conclusion, the fight against antibiotic resistance must be addressed as a global health emergency. This review aims to highlight the importance of finding strategies against this fight not only to find effective antimicrobials for the treatment of bacterial infections but also to protect the microbial balance.

Accumulating evidence indicates that this dysbiosis can trigger systemic inflammatory responses, increase intestinal permeability, and alter bidirectional communication along the gut-microbiota–brain axis. This imbalance can contribute to neuroinflammation, triggering pathological responses such as mood disorders, cognitive impairment, and neurodegeneration. However, it is important to note that many of these neurobiological effects remain to be directly confirmed in human studies. In this perspective, understanding the relationship between microbial health and brain function remains a critical research priority. Future studies are needed to identify new therapeutic strategies aimed at preserving microbial biodiversity, which is essential for both neurological and systemic health.

## Figures and Tables

**Table 1:** This table explains how antibiotic resistance spreads among bacterial populations through different genetic mechanisms.

Transmission Type	Mechanism	Description	Involved Elements	Example	Effects	Refs.
**Vertical**	Hereditary Mutations	Random mutations that confer resistance are passed down to bacterial progeny during cell division.	Chromosomal DNA mutations	Mutations in the *gyrA* and *parC* genes.	Confer resistance to fluoroquinolones by altering topoisomerases and reducing antibiotic binding affinity.	[23]
			Efflux pump genes (*AcrAB-TolC*)	Overexpression of *AcrAB-TolC* efflux system	Increased efflux of antibiotics, contributing to MDR.	[25]
			Porin genes (*OmpF*, *OmpC*) and Regulatory pathways (σE cycle)	Mutations reducing expression of OmpF/OmpC and altering σE stress response system.	Reduced membrane permeability, limiting antibiotic uptake and modulation of resistance and susceptibility profiles.	[26]
**Horizontal**	Conjugation	Direct transfer of plasmids containing resistance genes between bacteria via the conjugative pilus.	Plasmids, conjugative pilus	*Escherichia coli* transferring ESBL resistance plasmids to another strain.	Rapid transfer of resistance traits between bacteria, leading to increased resistance across different species.	[29,30]
	Transformation	Uptake of free DNA fragments from the environment by competent bacteria.	Free DNA, competent bacteria	Transformation of *Streptococcus pneumoniae* with DNA from a resistant strain.	Acquisition of resistance genes from the environment, leading to new resistant strains.	[32]
	Transduction	Transfer of resistance genes mediated by bacteriophages that infect bacteria.	Bacteriophages, bacterial DNA	Transfer of beta-lactamase genes by bacteriophage infection in *Pseudomonas aeruginosa*.	Genes from bacteriophages can integrate into bacterial genomes, spreading resistance in previously susceptible strains.	[40,41]
	Transposition	Movement of resistance genes within the genome or between plasmids and chromosomes via transposons.	Transposons, chromosomal and plasmid DNA	Transfer resistance genes via transposon in *Enterococcus faecalis* and *Enterococcus faecium*.	Facilitates the spread of resistance genes both within the bacterial chromosome and between plasmids.	[43,44]
	Integrons	Integration of gene cassettes containing resistance genes into MGEs.	Integrons, gene cassettes	Class 1 integrons in *Enterobacteriaceae* incorporating aminoglycoside resistance genes.	Enables bacteria to capture and integrate resistance genes from various sources, spreading resistance across species.	[46,47]

MDR: multidrug resistance; ESBL: extended-spectrum beta-lactam; MGEs: Mobile Genetic Elements; *gyrA*: *DNA gyrase subunit A*; parC: *Partition gene C*; *AcrAB-TolC*: Acriflavine Resistance AB channel protein.

**Table 2:** Summary of the effects of antibiotics and related microbial conditions on neuroinflammation and neurodegeneration. This table summarizes the key observed effects in animals following antibiotic-induced dysbiosis, highlighting the molecular mechanisms involved, the animal models used, the physiological and behavioral effects, and the implications for neurodegenerative diseases and behavioral changes. References for each study cited are provided.

Antibiotic/ Condition	Model/ Species	Mechanism of Action	Observed Effect (Behavior/Physiology)	Disease Stage	Refs.
Antibiotic-induced microbiota depletion	Rodents (general)	Increased LPS levels triggering immune activation	Neuroinflammation; behavioral changes	General/Induced dysbiosis	[134]
LPS exposure	Rodents (general)	Increased gut permeability via epithelial damage; systemic inflammation	Neuroinflammation; chronic systemic inflammation	General/Induced dysbiosis	[13,136]
Antibiotic-induced dysbiosis (hens)	Laying hens	Restoration of serotonin metabolism; microbiota balance	Reduced stress-related behaviors	Antibiotic-induced dysbiosis	[140,141]
Secretome of Akkermansia muciniphila	Enteroendocrine cells (in vitro)	Promotion of α-syn aggregation	α-syn aggregation linked to PD pathogenesis	Pathogenesis of PD	[142]
*Bacteroides ovatus* metabolites	Commensal gut bacteria	Inhibition of α-syn aggregation via phenolic acids	Neuroprotective effect via decreased aggregation	Potential therapeutic effect	[143]
*Bacillus subtilis* metabolites	Commensal gut bacteria	Inhibition of protein aggregation via sphingolipid modulation	Neuroprotective effect via decreased aggregation	Potential therapeutic effect	[144]
Germ-free state in AD mouse model	Mouse model (AD)	Reduced Aβ load; altered neuroinflammation	Reduced AD pathology and neuroinflammation	AD	[146]
Antibiotics in 5XFAD AD model	Mouse model (5XFAD Alzheimer’s)	Reduced pro-inflammatory microglia activation; decreased Aβ aggregation	Improved cognition and decreased neuroinflammation	AD	[147]
Infection in Pink1-KO PD model	Mouse model (Pink1-KO Parkinson’s)	Immune activation and recruitment of CD8+ T cells; increased butyric acid	Dopaminergic neuronal dysfunction; motor deficits	PD	[148]

LPS: Lipopolysaccharide, α-syn: Alpha-Synuclein, Aβ: Beta-Amyloid, AD: Alzheimer’s Disease, 5XFAD: A transgenic mouse model of AD (five familial AD mutations), PD: Parkinson’s Disease.

## Data Availability

Not applicable.

## Abbreviations

The following abbreviations are used in this manuscript:

ARGs	Antibiotic Resistance Genes
*GyrA*	*DNA gyrase subunit A*
*ParC*	*Partition gene C*
MDR	Multidrug Resistance
*AcrAB-TolC*	Acriflavine Resistance AB channel protein
ESBL	Extended-spectrum beta-lactam
CTX-M	Cefotaximase-Munich
IS	Insertion sequences
WHO	World Health Organization
*Cfx*A	*Cephalosporinase gene A*
*CepA*	*Cephalosporinase of Bacteroides*
*CblA*	*Cephalosporinase of Bacteroides Lineage A*
KPC-2	*Klebsiella pneumoniae* carbapenemase-2
NDM-1	New Delhi metal-β-lactamase-1
MRSA	Methicillin-resistant *Staphylococcus aureus*
*MCR*-1	Mobilized colistin resistance-1
MGEs	Mobile Genetic Elements
FDA	Food and Drug Administration
*TetM*	Tetracycline resistance gene M
*TetW*	Tetracycline resistance gene W
*TetQ*	Tetracycline Resistance Gene Q
IBD	Inflammatory bowel disease
CNS	Central Nervous System
ENS	Enteric Nervous System
PD	Parkinson’s Disease
ADHD	Attention-Deficit/Hyperactivity Disorder
GABA	γ-aminobutyric acid
SCFAs	Short-chain fatty acids
LPS	lipopolysaccharides
Aβ	beta-amyloid
BBB	Blood–brain barrier
